# Supplementary figures and images for: Cavin3 released from caveolae interacts with BRCA1 to regulate the cellular stress response
Source: eLife. 2021 Jun 18;10:e61407. doi: 10.7554/eLife.61407 (PMC8279762; doi:10.7554/eLife.61407)

**Figure 1-figure supplement 1-source data 1.**

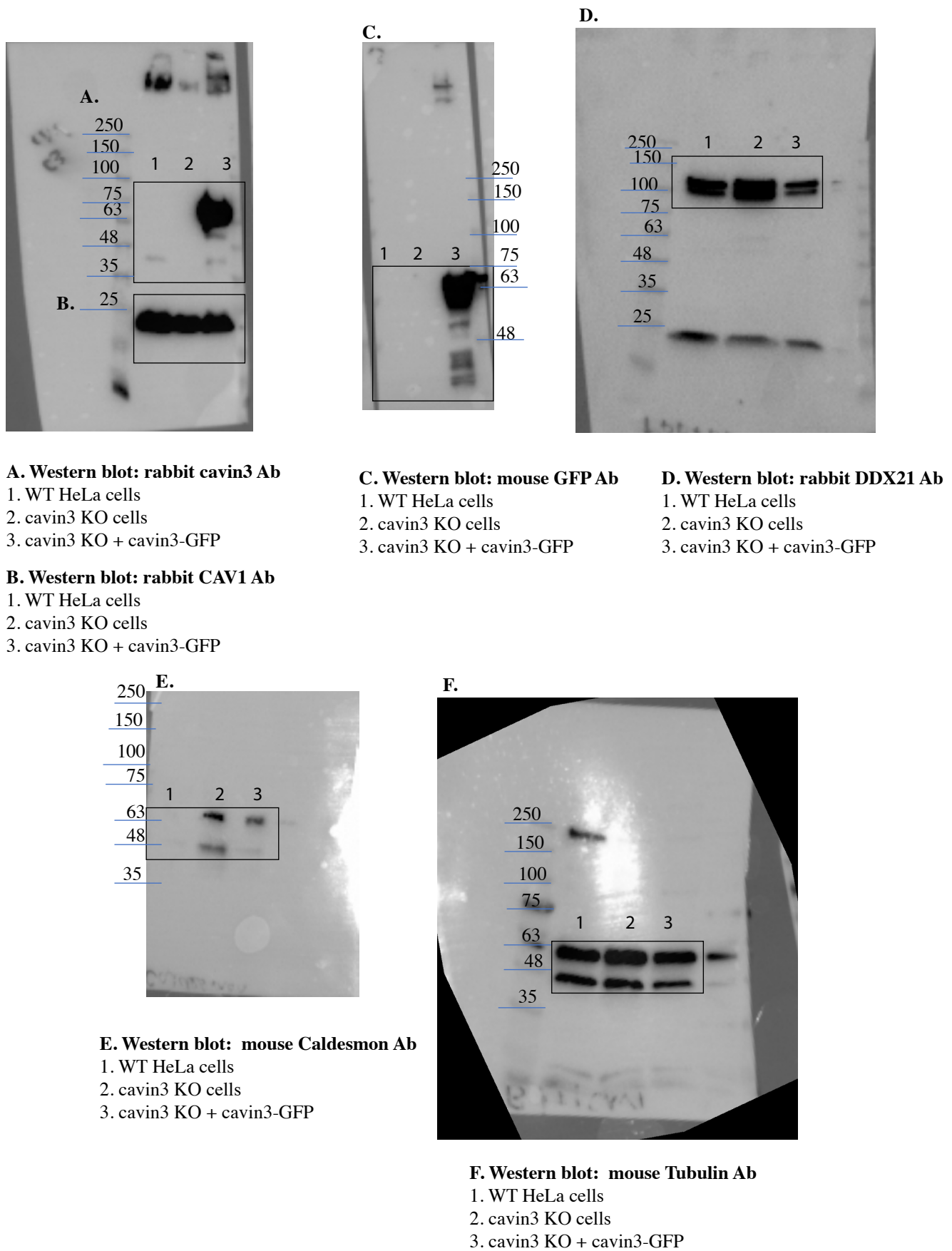

Supplement: Figure 1—figure supplement 1—source data 1. — (A) Western blot analysis of anti-rabbit cavin3, (B) anti-rabbit CAV1, (C) anti-rabbit ACLY, (D) anti-mouse alpha-catenin, (E) anti-rabbit ACCA, and (F) anti-rabbit EGFR antibodies in (1) WT HeLa cells and (2) cavin3 KO cells. [file elife-61407-fig1-figsupp1-data1.pdf]

Figure 1-figure supplement 1-source data 2

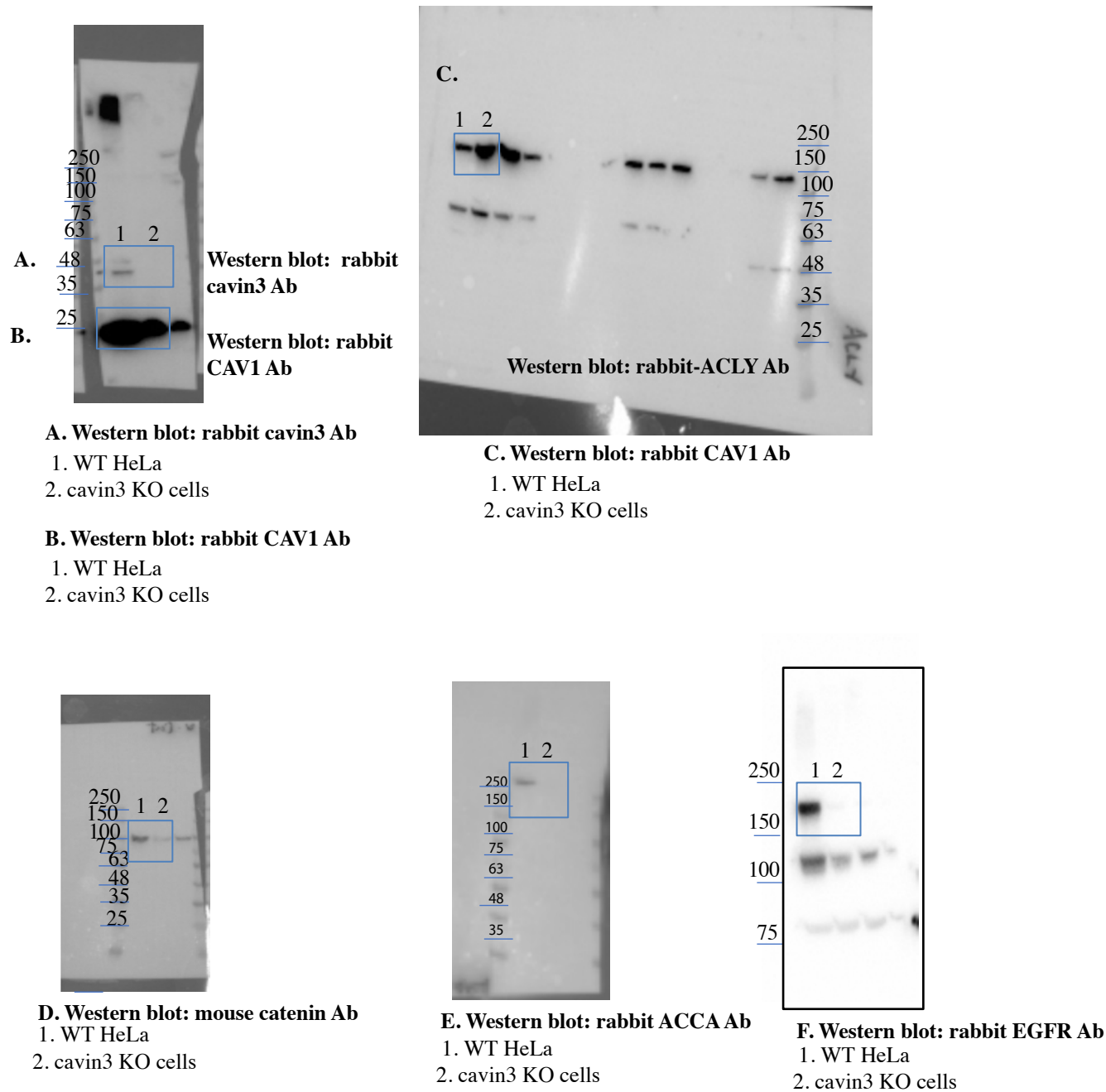

Supplement: Figure 1—figure supplement 1—source data 2. — (A) Western blot analysis of anti-rabbit cavin3, (B) anti-rabbit CAV1, (C) anti-mouse GFP, (D) anti-rabbit DDX21, (E) anti-rabbit Caldesmon and (F) anti-Tubulin antibodies in (1) HeLa WT, (2) cavin3 KO cells, and (3) cavin3KO + cavin3 GFP-expressing cells. [file elife-61407-fig1-figsupp1-data2.pdf]

Figure 4-source data 2.

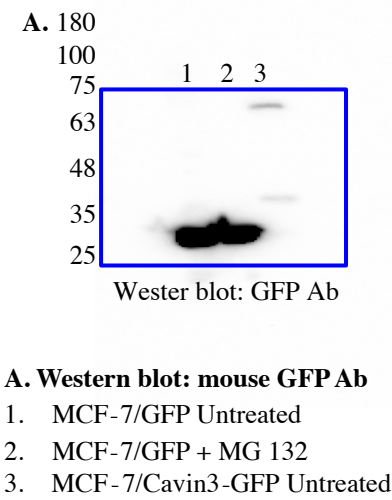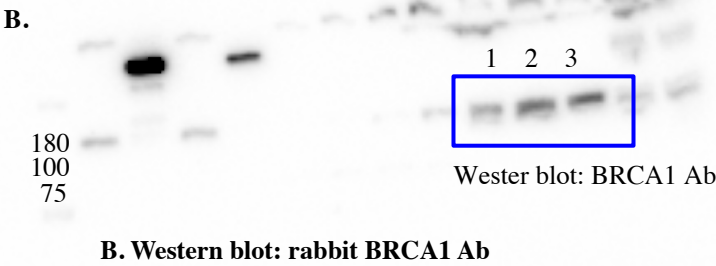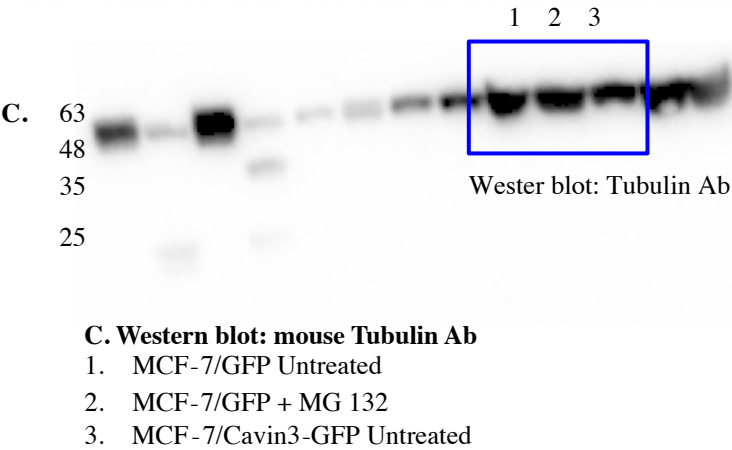

Supplement: Figure 4—source data 2. — (A) Western blot analysis of anti-mouse GFP, (B) anti-rabbit BRCA1, and (C) anti-mouse Tubulin antibodies in (1) MCF7/GFP untreated, (2) MCF7/GFP + MG132-treated lysates, and (3) MCF7/cavin3-GFP untreated lysates. [file elife-61407-fig4-data2.pdf]

**Figure 4-figure supplement 2-source data 1.**

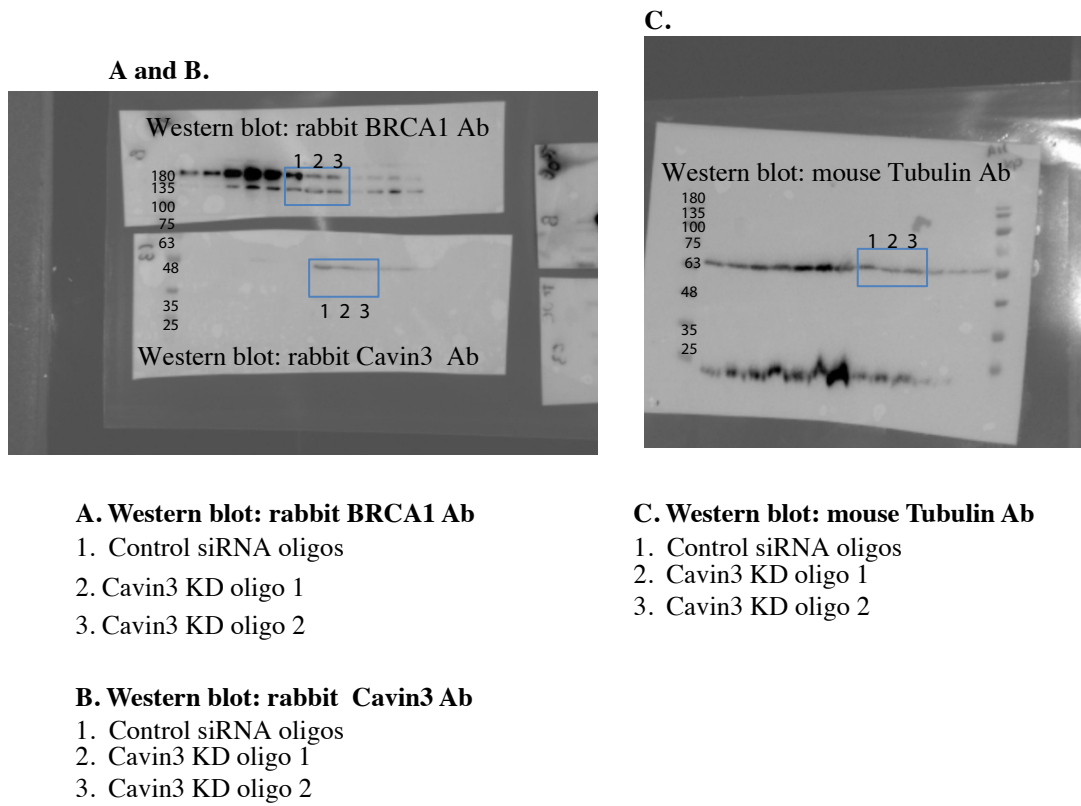

Supplement: Figure 4—figure supplement 2—source data 1. — (A) Western blot analysis of anti-rabbit BRCA1, (B) anti-rabbit cavin3, and (C) anti-mouse Tubulin in (1) MDA-MB231 treated with control siRNAs, (2) MDA-MB231 cells treated with cavin3-specific siRNA oligo 1, and (3) MDA-MB231 cells treated with cavin3-specific siRNA oligo 2. [file elife-61407-fig4-figsupp2-data1.pdf]

Figure 4-figure supplement 2-source data 2.

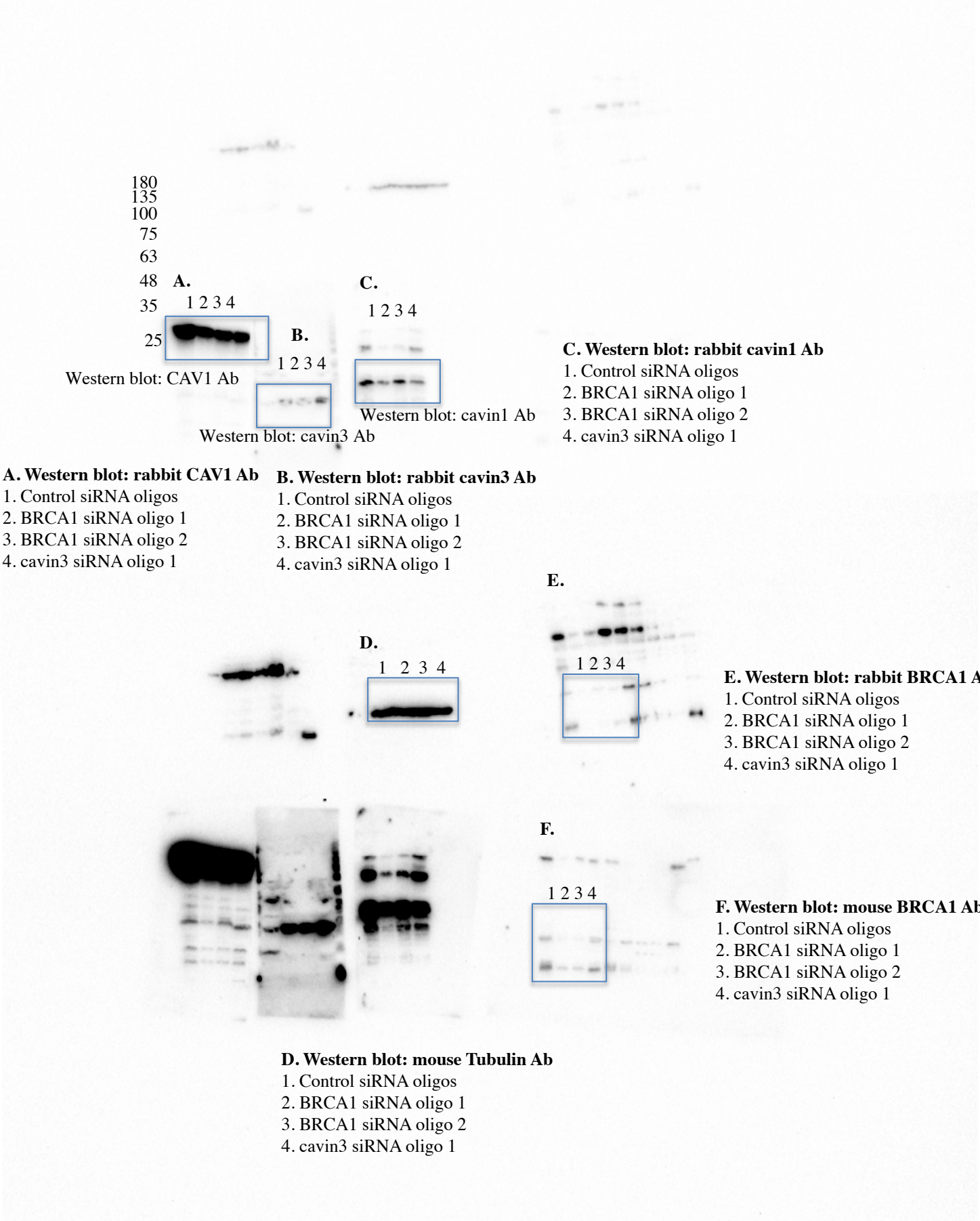

Supplement: Figure 4—figure supplement 2—source data 2. — (A) Western blot analysis of anti-rabbit CAV1, (B) anti-rabbit cavin3, (C) anti-rabbit cavin1, (D) anti-mouse Tubulin, (E) anti-rabbit BRCA1, and (F) anti-mouse BRCA1 in (1) MDA-MB231 cells treated with control siRNAs, (2) MDA-MB231 cells treated with BRCA1-specific siRNA oligo 1, (3) MDA-MB231 cells treated with BRCA1-specific siRNA oligo, and (4) MDA-MB231 cells treated with cavin3-specific oligo 1. [file elife-61407-fig4-figsupp2-data2.pdf]

Figure 8-figure supplement 2-source data 1.

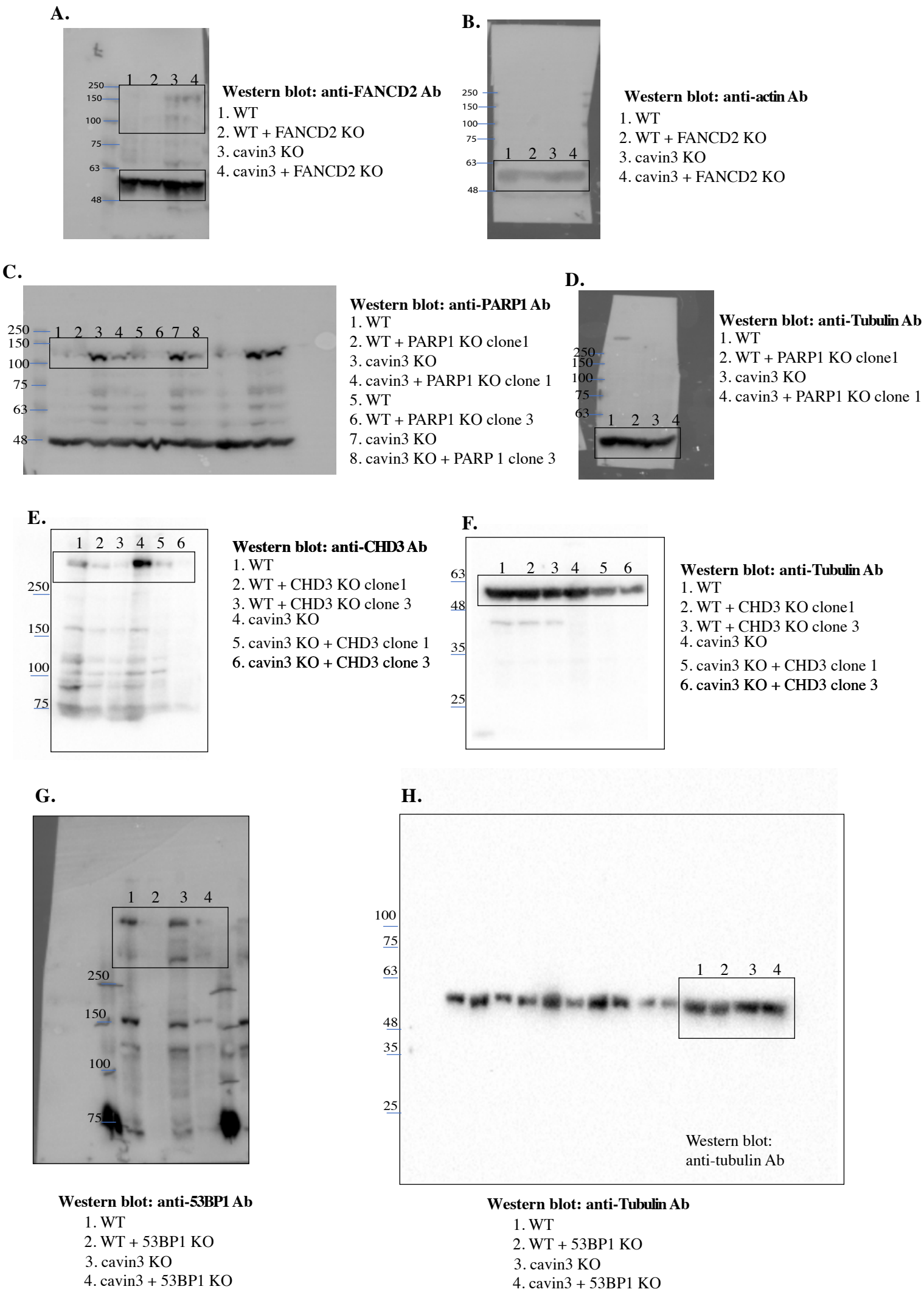

Supplement: Figure 8—figure supplement 2—source data 1. — (A) Western blot analysis of anti-rabbit FANCD2 and (B) anti-mouse actin antibodies in (1) WT HeLa, (2) WT HeLa + FANCD2 KO, (3) cavin3 KO, and (4) cavin3 KO + FANCD2 KO cells. (C) Western blot analysis of anti-rabbit PARP1 and (D) anti-mouse Tubulin antibodies in (1) WT HeLa, (2) WT HeLa + PARP1 KO clone 1, (3) cavin3 KO, (4) cavin3 KO + PARP1 KO clone 1, (5) WT HeLa, (6) WT + PARP1 KO clone 3, (7) cavin3 KO, and (8) cavin3 KO + PARP1 clone 3 cells. (E) Western blot analysis of anti-rabbit CHD3 and (F) anti-mouse Tubulin antibodies in (1) WT HeLa, (2) WT + CHD3 KO clone 1, (3) WT + CHD3 KO clone 3, (4) cavin3 KO cells, (5) cavin3 KO + CHD3 clone 1, and (6) cavin3 KO + CHD3 clone 3. (G) Western blot analysis of anti-rabbit 53BP1 and (H) anti-mouse Tubulin antibodies in (1) WT HeLa, (2) WT + 53BP1 KO, (3) cavin3 KO, and (4) cavin3 KO + 53BP1 KO cells. [file elife-61407-fig8-figsupp2-data1.pdf]
