# Supplementary material for: Cavin3 released from caveolae interacts with BRCA1 to regulate the cellular stress response
Source: eLife. 2021 Jun 18;10:e61407. doi: 10.7554/eLife.61407 (PMC8279762; doi:10.7554/eLife.61407)
Supplement: Figure 1—figure supplement 1—source data 3. — (A) Western blot analysis of anti-rabbit cavin3, (B) anti-rabbit CAV1, (C) anti-mouse GFP, (D) anti-rabbit DDX21, (E) anti-rabbit Caldesmon, and (F) anti-Tubulin antibodies in (1) HeLa WT, (2) cavin3 KO cells, and (3) cavin3KO + cavin3 GFP-expressing cells. [file elife-61407-fig1-figsupp1-data3.pdf]

Figure 1-figure supplement 1-source data 3.

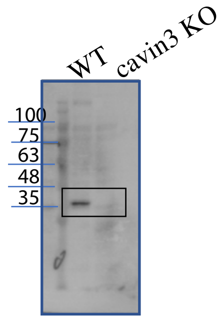

A. Western blot:
rabbit cavin3 Ab
1. WT HeLa cells
2. cavin3 KO cells

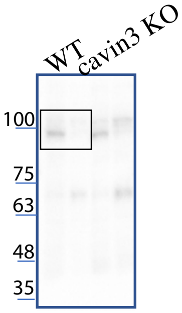

B. Western blot:
rabbit Rap80 Ab
1. WT HeLa cells
2. cavin3 KO cells

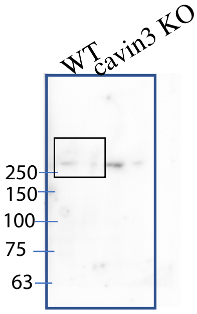

C. Western blot:
rabbit BRCA1 Ab
1. WT HeLa cells
2. cavin3 KO cells

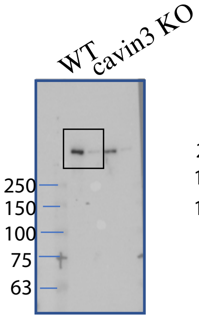

D. Western blot:
rabbit BRCA1 Ab
1. WT HeLa cells
2. cavin3 KO cells

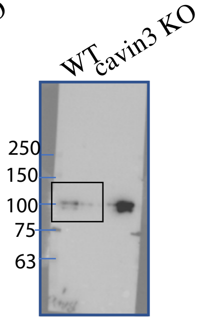

E. Western blot:
mouse BARD1Ab
1. WT HeLa cells
2. cavin3 KO cells

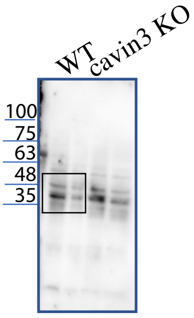

F. Western blot:
sheep Merit40 Ab
1. WT HeLa cells
2. cavin3 KO cells

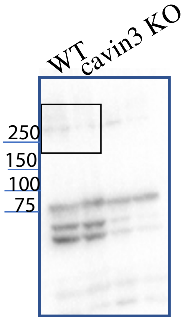

G. Western blot:
rabbit MDC1 Ab
1. WT HeLa cells
2. cavin3 KO cells

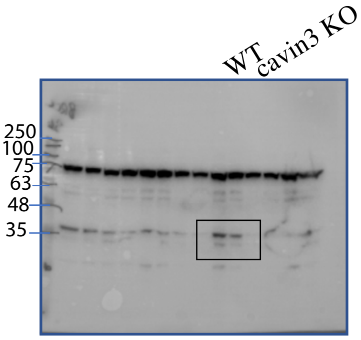

H. Western blot:
rabbit BRCC36 Ab
1. WT HeLa cells
2. cavin3 KO cells

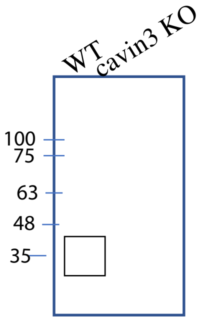

I. Western blot:
rabbit Rad51 Ab
1. WT HeLa cells
2. cavin3 KO cells

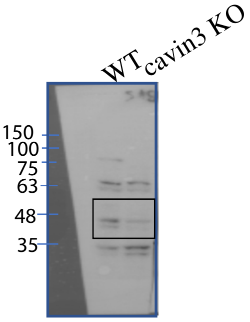

J. Western blot:
rabbit BRCC45 Ab
1. WT HeLa cells
2. cavin3 KO cells

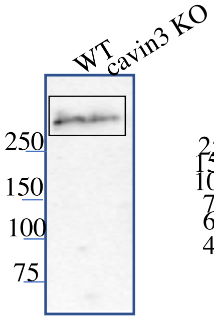

K. Western blot:
rabbit 53BP1 Ab
1. WT HeLa cells
2. cavin3 KO cells

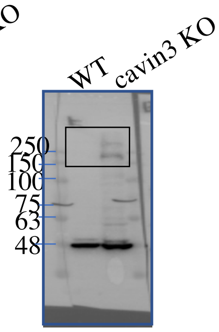

L. Western blot:
mouse FANCD2 Ab
1. WT HeLa cells
2. cavin3 KO cells

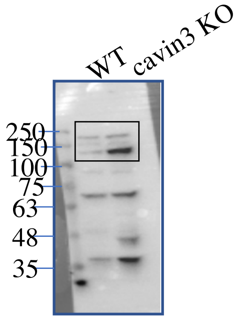

M. Western blot:
mouse HLTF Ab
1. WT HeLa cells
2. cavin3 KO cells

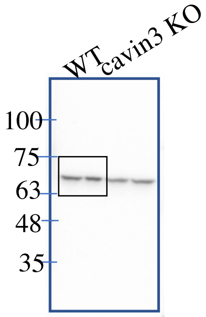

N. Western blot:
mouse Actin Ab
1. WT HeLa cells
2. cavin3 KO cells
