# Supplementary material for: Cavin3 released from caveolae interacts with BRCA1 to regulate the cellular stress response
Source: eLife. 2021 Jun 18;10:e61407. doi: 10.7554/eLife.61407 (PMC8279762; doi:10.7554/eLife.61407)
Supplement: Figure 4—source data 1. — (A) Western blot analysis of anti-rabbit BRCA1, (B) anti-mouse Tubulin, and (C) anti-mouse GFP antibodies in (1) GFP lysates, (2) cavin1-GFP lysates, and (3) cavin3-GFP lysates. [file elife-61407-fig4-data1.pdf]

Figure 4-source data 1.

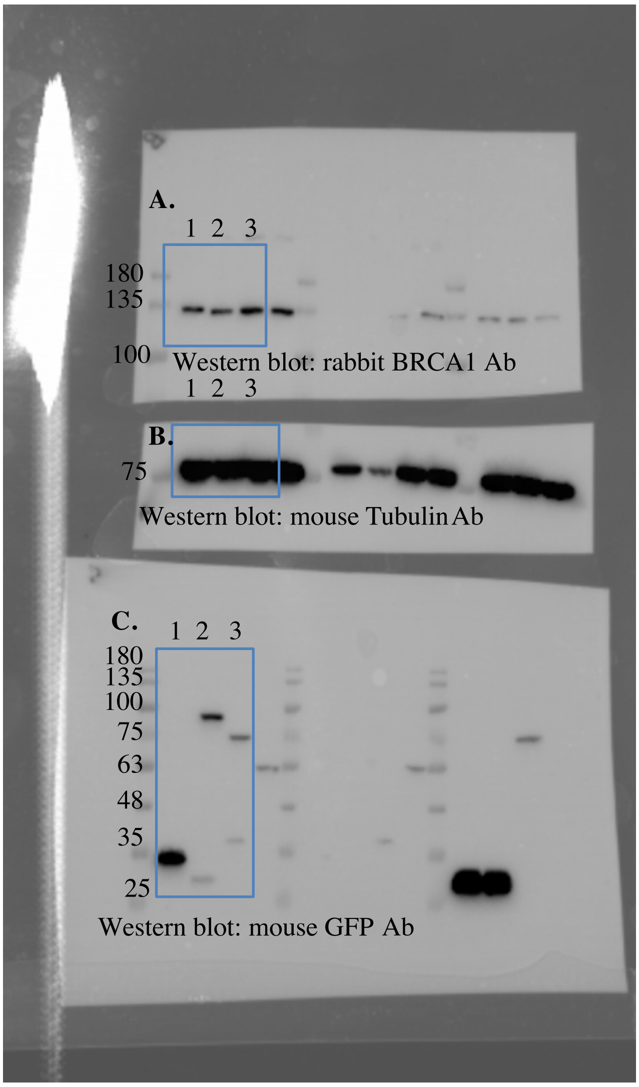

A. Western blot: rabbit BRCA1 Ab

1. GFP lysate
2. Cavin1⁻GFP lysate
3. Cavin3⁻GFP lysate

B. Western blot: mouse Tubulin Ab

1. GFP lysate
2. Cavin1⁻GFP lysate
3. Cavin3⁻GFP lysate

C. Western blot: mouse GFP Ab

1. GFP lysate
2. Cavin1⁻GFP lysate
3. Cavin3⁻GFP lysate

-
