# Supplementary material for: Cavin3 released from caveolae interacts with BRCA1 to regulate the cellular stress response
Source: eLife. 2021 Jun 18;10:e61407. doi: 10.7554/eLife.61407 (PMC8279762; doi:10.7554/eLife.61407)
Supplement: Figure 4—source data 3. — (A) Western blot analysis of anti-rabbit cavin3, (B) anti-rabbit CAV1, (C) anti-mouse Tubulin, and (D) anti-rabbit BRCA1 antibodies in (1) A431 cells treated with control siRNA oligos, (2) A431 cells treated with cavin3-specific siRNA oligo 1, and (3) A431 cells treated with cavin3-specific siRNA oligo 2. [file elife-61407-fig4-data3.pdf]

Figure 4-source data 3.

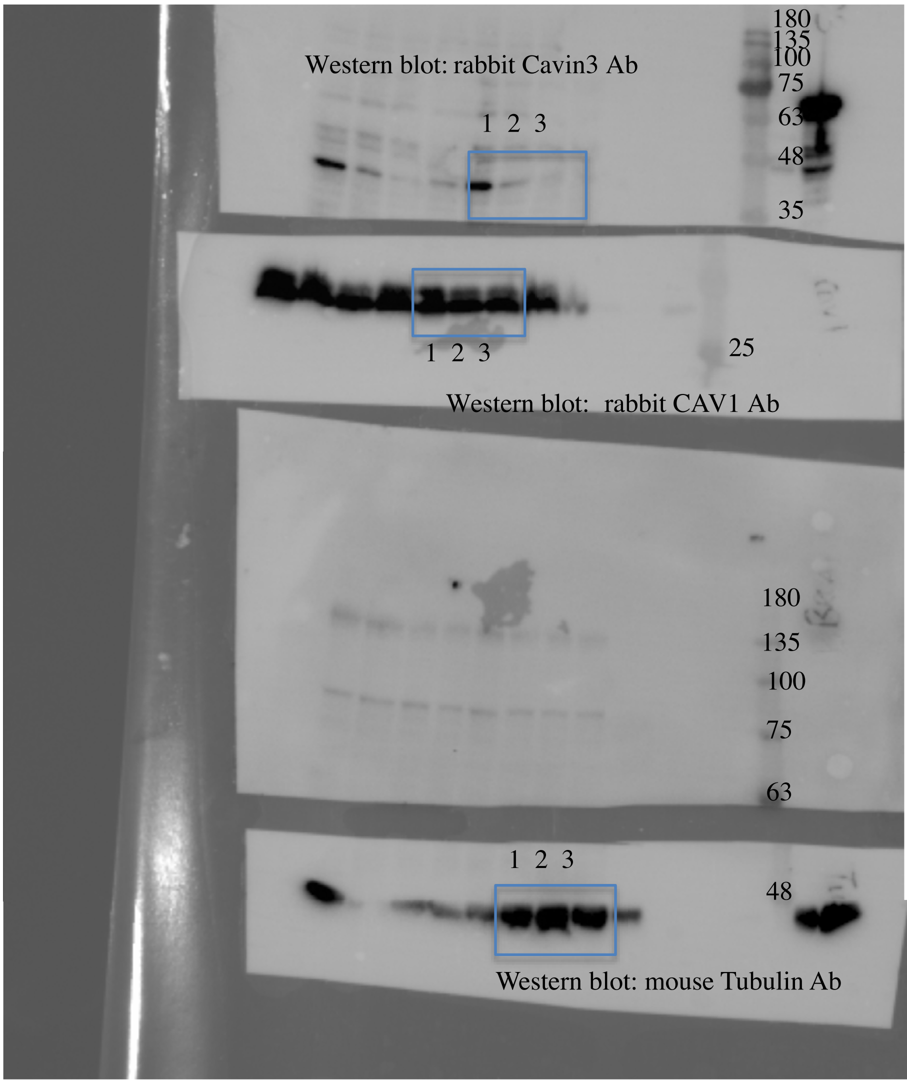

A. Western blot: rabbit Cavin3 Ab
1. Control siRNA oligos
2. Cavin3 siRNA oligo 1
3. Cavin3 siRNA oligo 2

B. Western blot: rabbit CAV1 Ab
1. Control siRNA oligos
2. Cavin3 siRNA oligo 1
3. Cavin3 siRNA oligo 2

Western blot: rabbit BRCA1 Ab (very weak)
1. Control siRNA oligos
2. Cavin3 siRNA oligo 1
3. Cavin3 siRNA oligo 2

C. Western blot: mouse Tubulin Ab
1. Control siRNA oligos
2. Cavin3 siRNA oligo 1
3. Cavin3 siRNA oligo 2

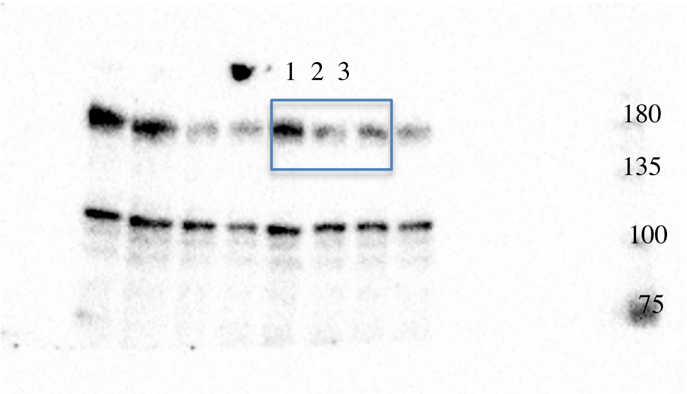

Western blot: rabbit BRCA1 Ab

D. Western blot: rabbit BRCA1 Ab
1. Control siRNA oligos
2. Cavin3 siRNA oligo 1
3. Cavin3 siRNA oligo 2
