# Supplementary material for: Cavin3 released from caveolae interacts with BRCA1 to regulate the cellular stress response
Source: eLife. 2021 Jun 18;10:e61407. doi: 10.7554/eLife.61407 (PMC8279762; doi:10.7554/eLife.61407)
Supplement: Figure 4—source data 4. — (A) Western blot analysis of anti-rabbit BRCA1, (B) anti-mouse Tubulin, (C) anti-rabbit cavin3, and (D) anti-rabbit CAV1 antibodies in A431 cells treated with (1) control siRNA oligos, (2) A431 cells treated with BRCA1-specific siRNA oligo 1, and (3) A431 cells treated with BRCA1-specific siRNA oligo 2. [file elife-61407-fig4-data4.pdf]

Figure 4-source data 4.

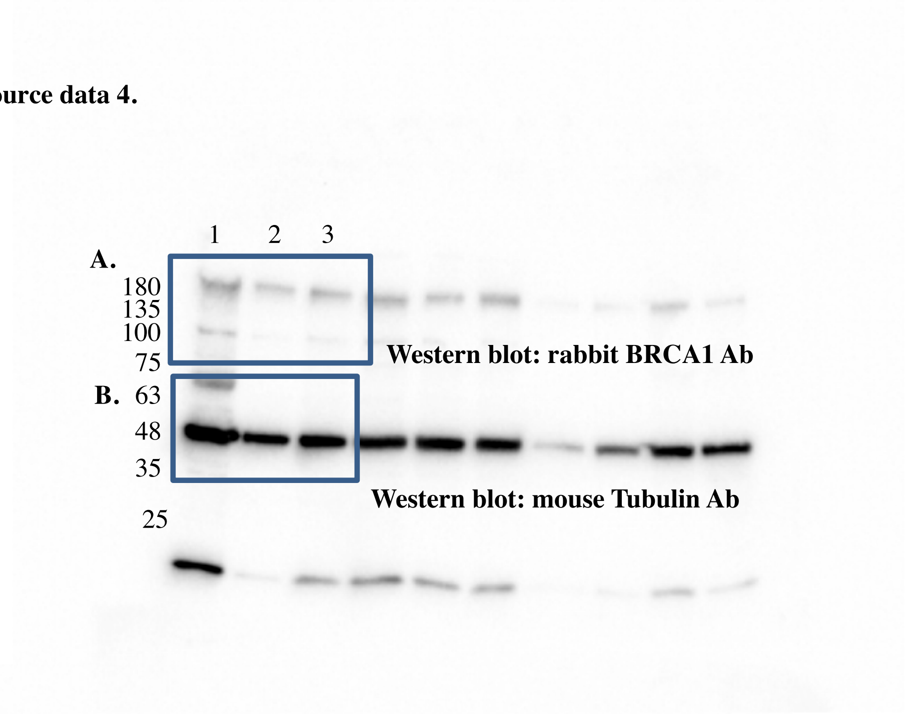

A41 cells

A. Western blot: rabbit BRCA1Ab

1. Control siRNA oligos
2. BRCA1 siRNA oligo 1
3. BRCA1 siRNA oligo 2

B. Western blot: mouse Tubulin Ab

1. Control siRNA oligos
2. BRCA1 siRNA oligo 1
3. BRCA1 siRNA oligo 2

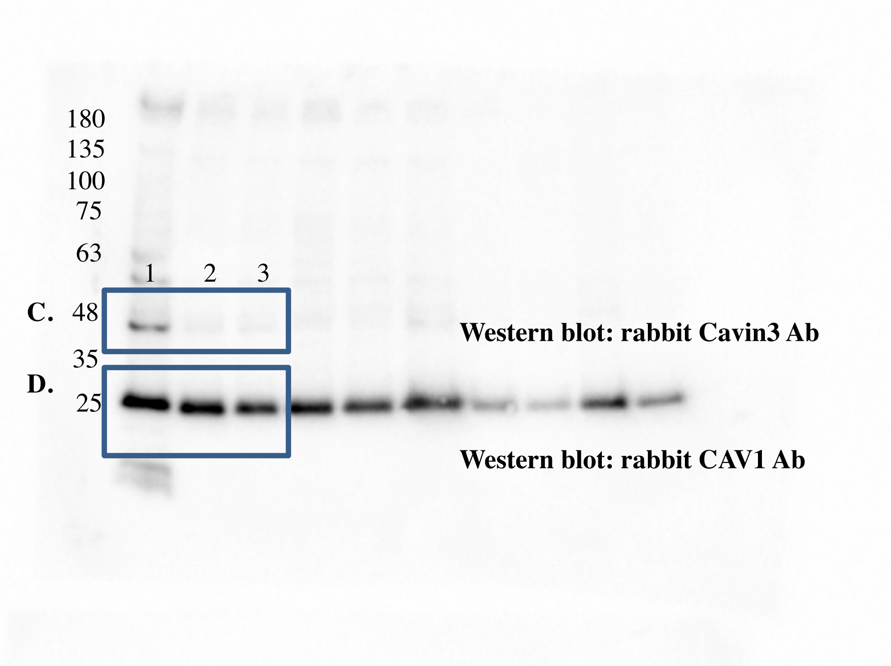

C. Western blot: rabbit Cavin3 Ab

1. Control siRNA oligos
2. BRCA1 siRNA oligo 1
3. BRCA1 siRNA oligo 2

D. Western blot: rabbit CAV1 Ab

1. Control siRNA oligos
2. BRCA1 siRNA oligo 1
3. BRCA1 siRNA oligo 2
