# Supplementary material for: Cavin3 released from caveolae interacts with BRCA1 to regulate the cellular stress response
Source: eLife. 2021 Jun 18;10:e61407. doi: 10.7554/eLife.61407 (PMC8279762; doi:10.7554/eLife.61407)
Supplement: Figure 4—source data 5. — (A) Western blot analysis of anti-rabbit BRCA1, (B) anti-rabbit cavin3, and (C) anti-mouse Tubulin antibodies in (1) A431 cells treated with control siRNA (control KD) oligos no treatment, (2) A431 cells treated with control siRNA oligos (control KD) and MG132 for 6 hr, (3) cavin3-specific siRNA (cavin3 KD) oligo 1 no treatment, (4) cavin3-specific siRNA (cavin3 KD) oligo 1 and MG132 for 6 hr, (5) cavin3-specific siRNA (cavin3 KD) oligo 2 no treatment, and (6) cavin3-specific siRNA (cavin3 KD) oligo 2 and MG132 for 6 hr. [file elife-61407-fig4-data5.pdf]

**Figure 4-source data 5.**

**A. Western blot: rabbit BRCA1 Ab**
1. Control KD no treatment
2. Control KD + MG132 6h
3. Cavin3 KD oligo 1 no treatment
4. Cavin3 KD oligo 1 + MG132 6h
5. Cavin3 KD oligo 2 no treatment
6. Cavin3 KD oligo 2 + MG132 6h

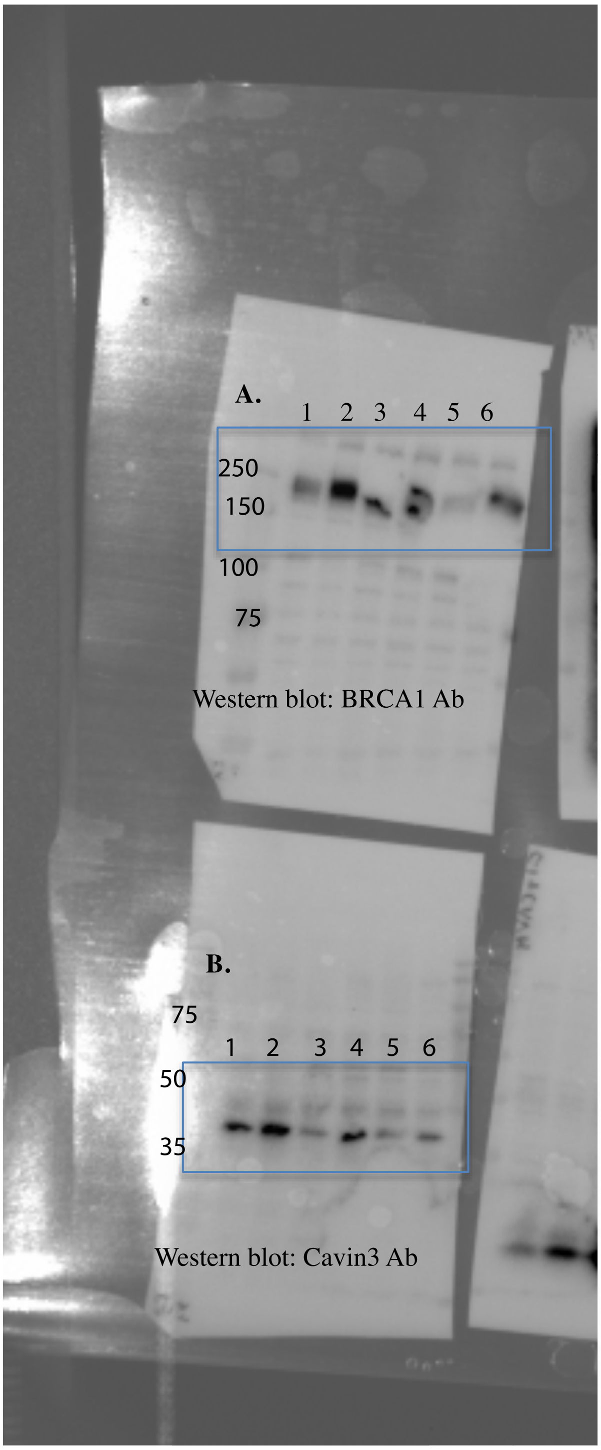


**B. Western blot: rabbit Cavin3 Ab**
1. Control KD no treatment
2. Control KD + MG132 6h
3. Cavin3 KD oligo 1 no treatment
4. Cavin3 KD oligo 1 + MG132 6h
5. Cavin3 KD oligo 2 no treatment
6. Cavin3 KD oligo 2 + MG132 6h

**A.** Western blot long exposure: BRCA1 Ab

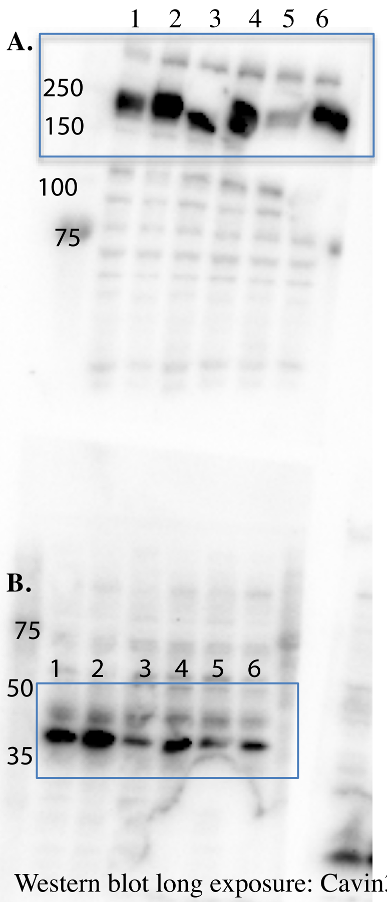


Western blot long exposure: Cavin3 Ab

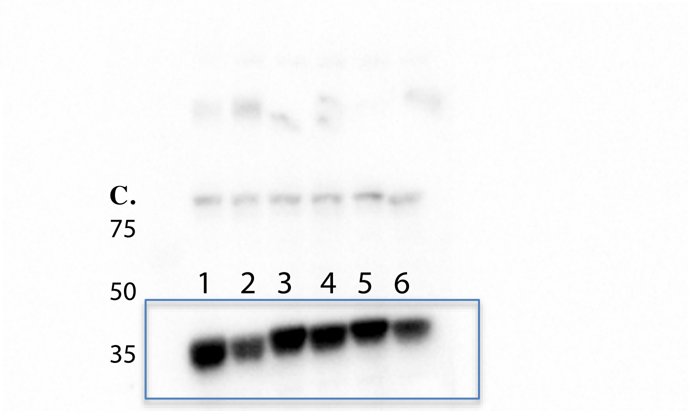


Western blot: Tubulin Ab

**C. Western blot: mouse Tubulin Ab**
1. Control KD no treatment
2. Control KD + MG132 6h
3. Cavin3 KD oligo 1 no treatment
4. Cavin3 KD oligo 1 + MG132 6h
5. Cavin3 KD oligo 2 no treatment
6. Cavin3 KD oligo 2 + MG132 6h
