# Supplementary material for: Cavin3 released from caveolae interacts with BRCA1 to regulate the cellular stress response
Source: eLife. 2021 Jun 18;10:e61407. doi: 10.7554/eLife.61407 (PMC8279762; doi:10.7554/eLife.61407)
Supplement: Figure 4—figure supplement 1—source data 1. — (A) Western blot analysis of anti-rabbit cavin3, (B) anti-mouse Tubulin, (C) anti-rabbit CAV1, and (D) anti-rabbit BRCA1 antibodies in (1) MCF7, (2) MDA-MB231, (3) A431, and (4) HeLa cells. [file elife-61407-fig4-figsupp1-data1.pdf]

Figure 4-figure supplement 1 source data 1.

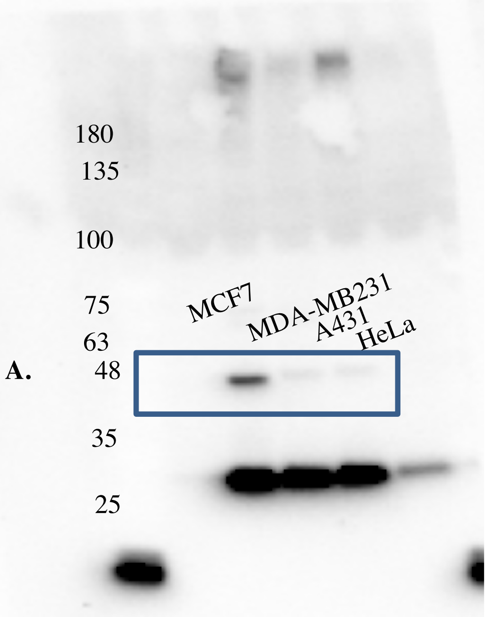

A. Western blot: mouse cavin3 Ab

1. MCF7 cells
2. MDA-MB231 cells
3. A431 cells
4. HeLa cells

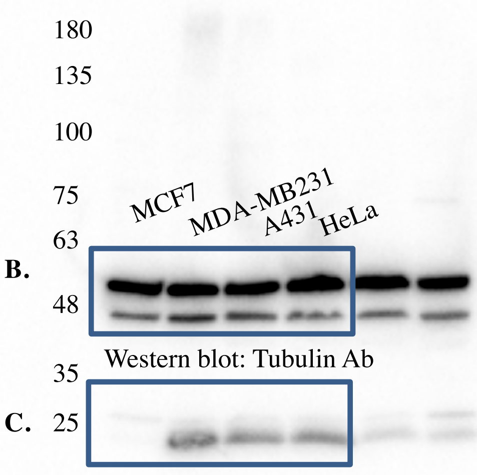

Western blot: CAV1 Ab

B. Western blot: mouse Tubulin Ab

1. MCF7
2. MDA-MB231
3. A431 cells
4. HeLa cells

C. Western blot: rabbit CAV1 Ab

1. MCF7 cells
2. MDA-MB231 cells
3. A431 cells
4. HeLa cells

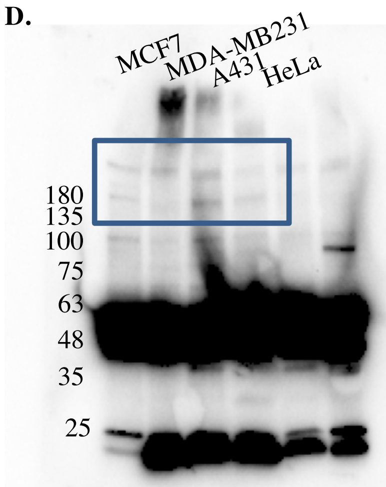

Western blot: BRCA1 Ab

D. Western blot: rabbit BRCA1 Ab

1. MCF7 cells
2. MDA-MB231 cells
3. A431 cells
4. HeLa cells
