# Supplementary material for: Cavin3 released from caveolae interacts with BRCA1 to regulate the cellular stress response
Source: eLife. 2021 Jun 18;10:e61407. doi: 10.7554/eLife.61407 (PMC8279762; doi:10.7554/eLife.61407)
Supplement: Figure 8—source data 1. — Western blot analysis of (A) anti-rabbit cavin3, (B) anti-rabbit CAV1, (C) anti-rabbit BRCA1, (D) anti-rabbit RAD51, and (E) anti-mouse Tubulin antibodies in (1) WT control, (2) WT UV 30 min chase, (3) WT UV 60 min chase, (4) WT UV 120 min chase, (5) WT UV 240 min chase, (6) cavin3 KO control, and (7) cavin3 KO UV 30 min chase, cavin3 KO UV 60 min chase, cavin3 KO UV 120 min chase, and cavin3 KO 240 min chase. [file elife-61407-fig8-data1.pdf]

Figure 8-source data 1.

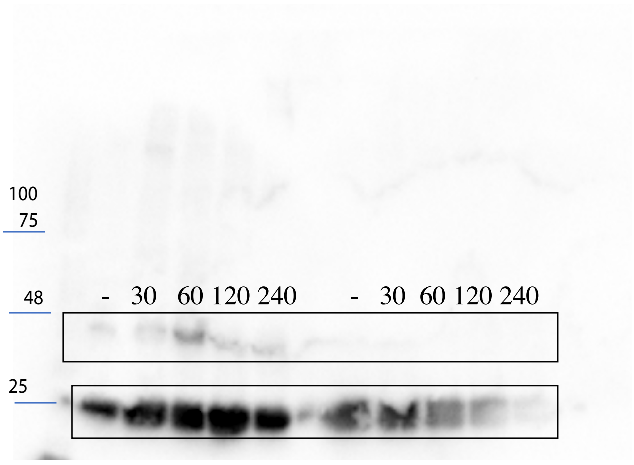

A and B. Western blot: rabbit cavin3 top and CAV1 bottom
1. WT control
2. WT UV 30 min chase
3. WT UV 60 min chase
4. WT UV 120 min chase
5. WT UV 240 min chase
6. cavin3 KO control
7. cavin3 KO UV 30 min chase
8. cavin3 KO UV 60 min chase
9. cavin3 KO UV 120 min chase
10. cavin3 KO UV 240 min chase

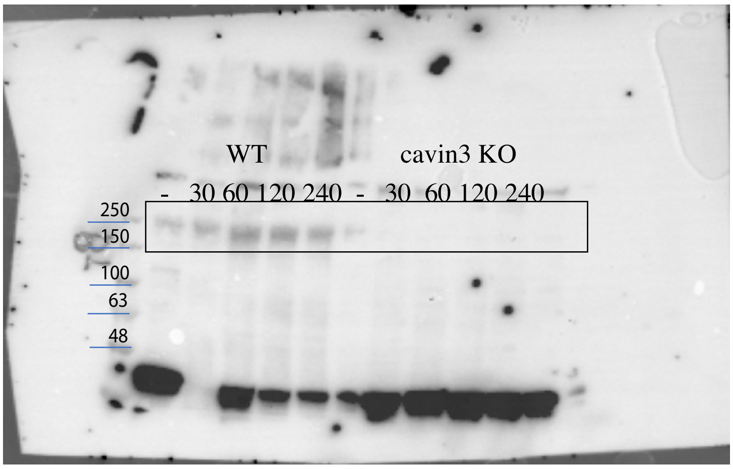

C. Western blot: rabbit BRCA1 Ab
1. WT control
2. WT UV 30 min chase
3. WT UV 60 min chase
4. WT UV 120 min chase
5. WT UV 240 min chase
6. cavin3 KO control
7. cavin3 KO UV 30 min chase
8. cavin3 KO UV 60 min chase
9. cavin3 KO UV 120 min chase
10. cavin3 KO UV 240 min chase

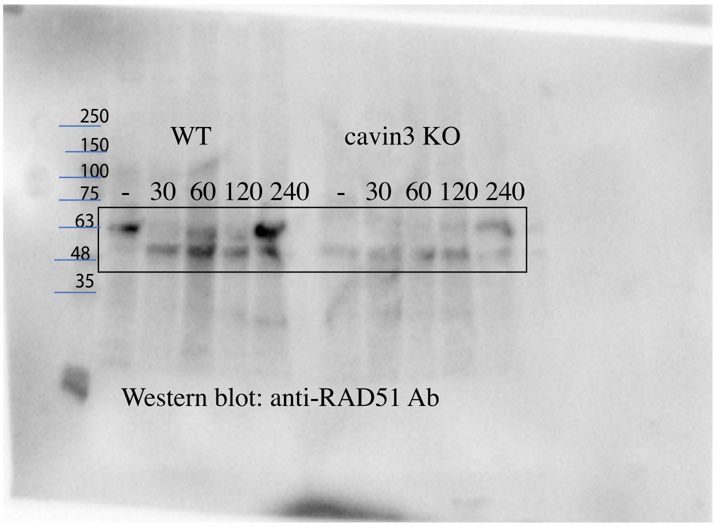

D. Western blot: rabbit Rad51 Ab
1. WT control
2. WT UV 30 min chase
3. WT UV 60 min chase
4. WT UV 120 min chase
5. WT UV 240 min chase
6. cavin3 KO control
7. cavin3 KO UV 30 min chase
8. cavin3 KO UV 60 min chase
9. cavin3 KO UV 120 min chase
10. cavin3 KO UV 240 min chase

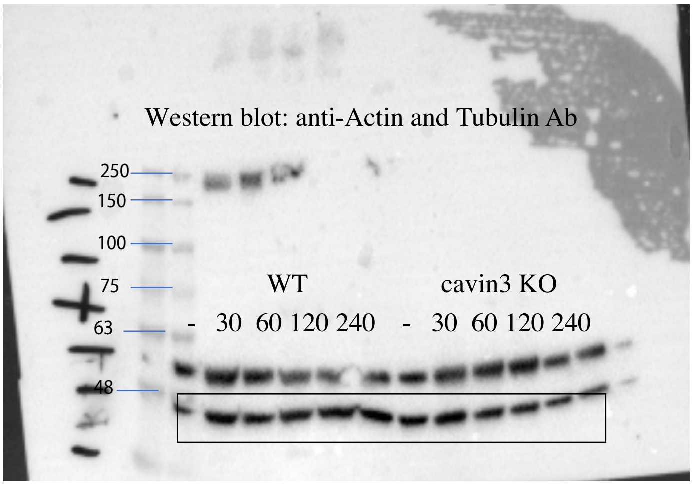

E. Western blot: mouse Tubuli (top) and Actin (bottom) Ab
1. WT control
2. WT UV 30 min chase
3. WT UV 60 min chase
4. WT UV 120 min chase
5. WT UV 240 min chase
6. cavin3 KO control
7. cavin3 KO UV 30 min chase
8. cavin3 KO UV 60 min chase
9. cavin3 KO UV 120 min chase
10. cavin3 KO UV 240 min chase
