# Supplementary material for: Cavin3 released from caveolae interacts with BRCA1 to regulate the cellular stress response
Source: eLife. 2021 Jun 18;10:e61407. doi: 10.7554/eLife.61407 (PMC8279762; doi:10.7554/eLife.61407)
Supplement: Figure 8—source data 2. — (A) Western blot analysis of anti-rabbit cavin3, (B) anti-rabbit BRCA1, (C) anti-rabbit Rad51 and γH2AX, (D) anti-rabbit BRCC36, (E) anti-sheep Merit40, (F) anti-rabbit BRCA2, (G) anti-rabbit CAV1, (H) anti-rabbit PKM, (I) anti-rabbit PGK1, and (J) anti-actin antibodies in (1) WT untreated cells, (2) WT + UV treatment and a 4 hr chase, (3) cavin3 KO cells, and (4) cavin3 KO + UV treatment and a chase 4 hr chase time. [file elife-61407-fig8-data2.pdf]

Figure 8-source data 2.

A.

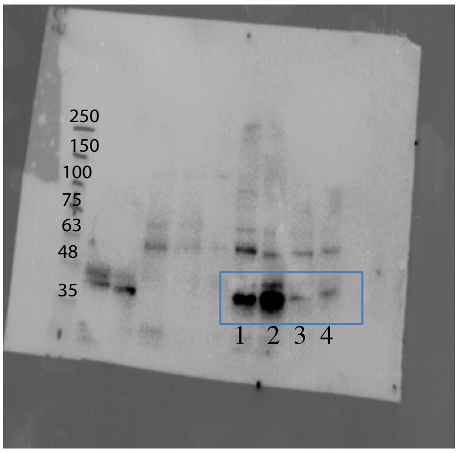

A. Western blot: rabbit cavin3 Ab
1. WT untreated
2. WT + UV treatment 4 hour chase
3. cavin3 KO untreated
4. cavin3 KO + UV treatment 4 hour chase

B.

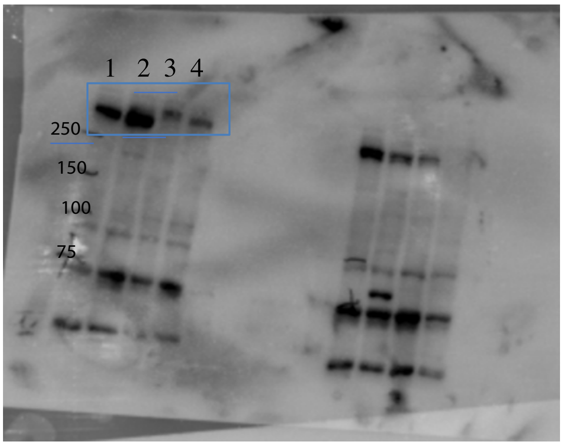

B. Western blot: rabbit BRCA1 Ab
1. WT untreated
2. WT + UV treatment 4 hour chase
3. cavin3 KO untreated
4. cavin3 KO + UV treatment 4 hour chase

C.

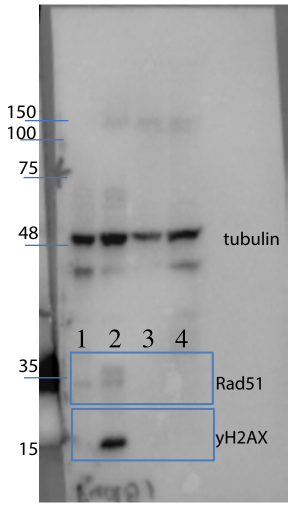

C. Western blot: rabbit Rad51 and yH2AX
1. WT untreated
2. WT + UV treatment 4 hour chase
3. cavin3 KO untreated
4. cavin3 KO + UV treatment 4 hour chase

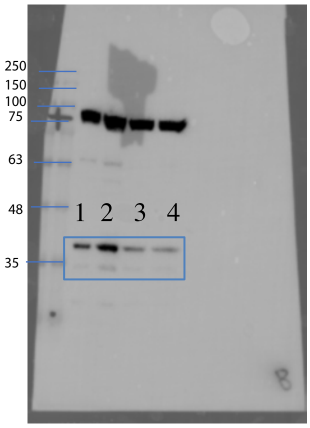

D. Western blot: rabbit BRCC36 Ab
1. WT untreated
2. WT + UV treatment 4 hour chase
3. cavin3 KO untreated
4. cavin3 KO + UV treatment 4 hour chase

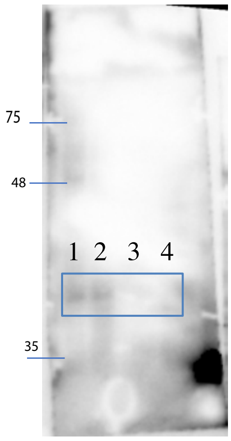

E. Western blot: sheep Merit 40Ab
1. WT untreated
2. WT + UV treatment 4 hour chase
3. cavin3 KO untreated
4. cavin3 KO + UV treatment 4 hour chase

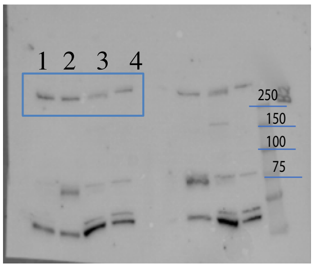

F. Western blot: rabbit BRCA2 Ab
1. WT untreated
2. WT + UV treatment 4 hour chase
3. cavin3 KO untreated
4. cavin3 KO + UV treatment 4 hour chase

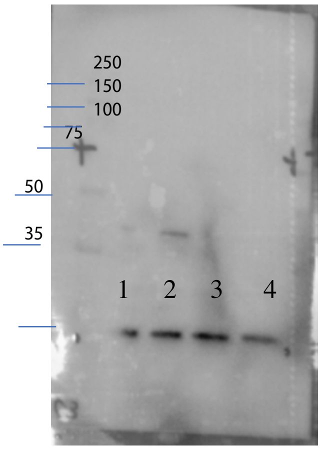

G. Western blot: rabbit CAV1 Ab
1. WT untreated
2. WT + UV treatment 4 hour chase
3. cavin3 KO untreated
4. cavin3 KO + UV treatment 4 hour chase

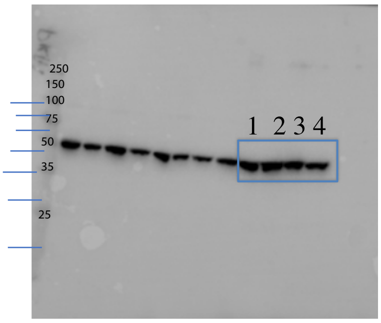

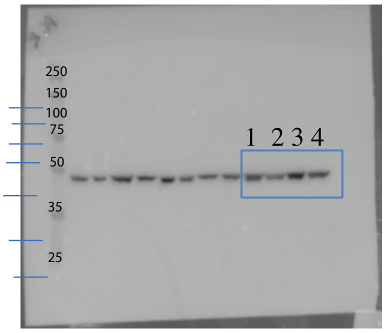

H and I. Western blot: rabbit PKM and PGK Ab
1. WT untreated
2. WT + UV treatment 4 hour chase
3. cavin3 KO untreated
4. cavin3 KO + UV treatment 4 hour chase

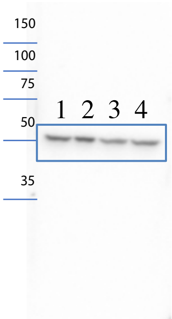

J. Western blot: mouse actin Ab
1. WT untreated
2. WT + UV treatment 4 hour chase
3. cavin3 KO untreated
4. cavin3 KO + UV treatment 4 hour chase
