# Supplementary material for: Cavin3 released from caveolae interacts with BRCA1 to regulate the cellular stress response
Source: eLife. 2021 Jun 18;10:e61407. doi: 10.7554/eLife.61407 (PMC8279762; doi:10.7554/eLife.61407)
Supplement: Supplementary file 3. [file elife-61407-supp3.docx]

***Supplementary File 3. Supplementary Discussion***

In addition to the newly defined roles in homologous recombination, cavin3 may play a role in replication fork reversal as several proteins in BRCA1-deficient cells such as Helicase like Transcription Factor (HLTF, ~2.3 log increase) are significantly upregulated in cavin3-deficient cells. Replication fork reversal is an important protective mechanism that allows forks to reverse their course when DNA lesions are encountered allowing for the resumption of DNA synthesis without chromosome breaks (Neelsen and Lopes, 2015, reviewed in Quinet et al., 2017). Several recent studies by Kolinjivadi et al., 2017; Taglialatela et al., 2017, Vulganovic et al., 2017 have demonstrated that the translocase activity of ZRANB3 and SMARCAL1 is required for replication fork reversion which is a newly identified function for BRCA1 and BRCA2. ZRANB3 was shown to interact with polyubiquitinated PCNA to promote fork remodeling. Similarly, HLTF was also demonstrated to have DNA translocase activity and to similarly promote PCNA polyubiquitination further implicating HLTF in replication fork reversal (Blastyak et al., 2010). Given the fact that recent studies have suggested that BRCA1 is a crucial regulator of replication fork degradation and that the mechanism leading to fork degradation in these cells remains unclear, further studies are warranted into how different fork remodelers such as ZRANB3 and HLTF work together in this process. As such, cavin3 KO cells may provide an alternative cell system for studying fork degradation in a cell line that is also deficient in BRCA1.

Cavin3 KO cells also upregulate proteins that promote the error-prone Pol/PARP1 mediated Alternate-End Joining (alt-EJ) DNA repair pathway; these included members of the Fanconi anemia (FA) pathway (FANCD2), Fanconi anemia protein component I (FANCI) as well as PARP1, TOPBP1, DROSHA, and RPA2 (**Figure 1**). FANCI was recently identified as a potential cavin3 interacting protein in previous studies from our laboratory (McMahon et al., 2019). These findings imply that cavin3 deficient cells may upregulate the error-prone Pol/PARP1 mediated alt-EJ DNA repair pathway to compensate for defective homologous recombination DNA repair (Ceccaldi et al., 2015; Mateos-Gomez et al., 2015). These findings support further evaluation of alternative repair pathways in cavin3 KO cells.

The minichromosome maintenance proteins, MCM2 to MCM7, form a heteromeric DNA helicase required for DNA replication licensing where it primes chromatin for DNA replication (reviewed by Tye et al., 1999; Giaginis et al., 2010). Although DNA helicase activity is required to establish a bidirectional replication fork from each replication origin, a large excess of MCM complexes is accumulated. The role of the additional MCM complexes is not well understood, as most is displaced from the DNA during S-phase, without playing an active role in DNA replication. Recent studies have demonstrated that MCM2-7 expression is downregulated in cells experiencing chronic replication stress and this depends on the tumor suppressor p53 (Bai et al., 2016). A decrease in both HR and NHEJ mediated repair pathways has been demonstrated in cells with a reduction of MCM complex proteins. It was shown that the MCM complex modulates the cellular response to DNA DSBs. Indeed, cavin3 KO cells also exhibit downregulation of the MCM proteins, MCM2-7, which may further contribute to the decrease in HR found in these cells. Collectively, these results emphasize the importance of appropriately balancing different repair pathways to maintain global genomic stability and to contribute to cell survival and suggest that cavin3 KO cells may be useful for studying these pathways which are particularly relevant in breast and ovarian cancer cells.

***Supplementary Information References***

Bai, G., Smolka, M.B., and Schimenti, J.C. (2016). Chronic DNA Replication Stress Reduces Replicative Lifespan of Cells by TRP53-Dependent, microRNA-Assisted MCM2-7 Downregulation. PLoS Genet ***12***, e1005787.

Blastyak, A., Hajdu, I., Unk, I., and Haracska, L. (2010). Role of double-stranded DNA translocase activity of human HLTF in replication of damaged DNA. Molecular and cellular biology ***30*,** 684-693.

Ceccaldi, R., Rondinelli, B., and D'Andrea, A.D. (2016). Repair Pathway Choices and Consequences at the Double-Strand Break. Trends Cell Biol ***26***, 52-64.

Giaginis, C., Giagini, A., Tsourouflis, G., Gatzidou, E., Agapitos, E., Kouraklis, G., and Theocharis, S. (2011). MCM-2 and MCM-5 expression in gastric adenocarcinoma: clinical significance and comparison with Ki-67 proliferative marker. Digestive diseases and sciences ***56***, 777-785.

Kolinjivadi, A.M., Sannino, V., De Antoni, A., Zadorozhny, K., Kilkenny, M., Techer, H., Baldi, G., Shen, R., Ciccia, A., Pellegrini, L.*, et al.* (2017). Smarcal1-Mediated Fork Reversal Triggers Mre11-Dependent Degradation of Nascent DNA in the Absence of Brca2 and Stable Rad51 Nucleofilaments. Mol Cell ***67***, 867-881 e867.

McMahon, K.A., Wu, Y., Gambin, Y., Sierecki, E., Tillu, V.A., Hall, T., Martel, N., Okano, S., Moradi, S.V., Ruelcke, J.E.*, et al.* (2019). Identification of intracellular cavin target proteins reveals cavin-PP1alpha interactions regulate apoptosis. Nature communications ***10***, 3279.

Neelsen, K.J., and Lopes, M. (2015). Replication fork reversal in eukaryotes: from dead end to dynamic response. Nat Rev Mol Cell Biol ***16***, 207-220.

Quinet, A., Lemacon, D., and Vindigni, A. (2017). Replication Fork Reversal: Players and Guardians. Mol Cell ***68***, 830-833.

Taglialatela, A., Alvarez, S., Leuzzi, G., Sannino, V., Ranjha, L., Huang, J.W., Madubata, C., Anand, R., Levy, B., Rabadan, R.*, et al.* (2017). Restoration of Replication Fork Stability in BRCA1- and BRCA2-Deficient Cells by Inactivation of SNF2-Family Fork Remodelers. Mol Cell ***68***, 414-430 e418.

Tye, B.K. (1999). MCM proteins in DNA replication. Annu Rev Biochem ***68*,** 649-686.

Vujanovic, M., Krietsch, J., Raso, M.C., Terraneo, N., Zellweger, R., Schmid, J.A., Taglialatela, A., Huang, J.W., Holland, C.L., Zwicky, K.*, et al.* (2017). Replication Fork Slowing and Reversal upon DNA Damage Require PCNA Polyubiquitination and ZRANB3 DNA Translocase Activity. Mol Cell ***67***, 882-890 e885.
