# Supplementary material for: Cavin3 released from caveolae interacts with BRCA1 to regulate the cellular stress response
Source: eLife. 2021 Jun 18;10:e61407. doi: 10.7554/eLife.61407 (PMC8279762; doi:10.7554/eLife.61407)
Supplement: Transparent reporting form [file elife-61407-transrepform.docx]

***eLife’s* transparent reporting form**

We encourage authors to provide detailed information *within their submission* to facilitate the interpretation and replication of experiments. Authors can upload supporting documentation to indicate the use of appropriate reporting guidelines for health-related research (see [EQUATOR Network](http://www.equator-network.org/%20)), life science research (see the [BioSharing Information Resource](https://biosharing.org/)), or the [ARRIVE guidelines](http://www.plosbiology.org/article/info:doi/10.1371/journal.pbio.1000412) for reporting work involving animal research. Where applicable, authors should refer to any relevant reporting standards documents in this form.

If you have any questions, please consult our Journal Policies and/or contact us: [editorial@elifesciences.org](mailto:editorial@elifesciences.org).

**Sample-size estimation**

- You should state whether an appropriate sample size was computed when the study was being designed
- You should state the statistical method of sample size computation and any required assumptions
- If no explicit power analysis was used, you should describe how you decided what sample (replicate) size (number) to use

Please outline where this information can be found within the submission (e.g., sections or figure legends), or explain why this information doesn’t apply to your submission:

For each figure panel where quantitation is involved, the legend describes the statistical test that was used, the number of cells or samples and the number of independent experiments the data was derived from.

We performed power analysis, where Alpha Error is at the level of 5% and Beta Error is at the level of 10 %, using average values and Standard Deviation of different samples from preliminary PLA quantitative results to determine the sample size. The calculation results suggested that the required sample size “N” is at least “1” for detecting statistical significance for all the PLA experiments in this paper.

The significance criterion i.e p < 0.0001 in many experiment conditions and sample size i.e 150 total cells analysed for three independent experiments for all the PLA experiments was determined to be give adequate representation of the variation within and between samples. The control and treated groups of cells came from the same cell cultures and were treated and analysed at the same time for each experiment.

**Replicates**

- You should report how often each experiment was performed
- You should include a definition of biological versus technical replication
- The data obtained should be provided and sufficient information should be provided to indicate the number of independent biological and/or technical replicates
- If you encountered any outliers, you should describe how these were handled
- Criteria for exclusion/inclusion of data should be clearly stated
- High-throughput sequence data should be uploaded before submission, with a private link for reviewers provided (these are available from both GEO and ArrayExpress)

Please outline where this information can be found within the submission (e.g., sections or figure legends), or explain why this information doesn’t apply to your submission:

**Figure 1 -** Three replicates from matched WT and cavin3 KO HeLa cells were analysed with a total of 4206 proteins were robustly quantified, detected with >2 unique peptides and an FDR <1.0 % in at least 2 out of 3 replicate as described in the results section and in the corresponding figure legend.

**Figure 2 and Figure 2-figure supplement 1-see Single molecule spectrometry in Material and Methods section for details –** more than 1000 events were collected in all cases.

**Figure 3F, 5E, 6E-H, Figure 3-figure supplement 1, Figure 6-figure supplement 1, Figure 8- figure supplement 2** see figure legend for details**-** Quantified PLA signals are represented as the number of red dots in 150 total cells (n=50 cells per experiment, three independent experiments.

**Figure 7A, D-F**, **Figure 7-figure supplement 1-** see figure legend for details - LDH release - was calculated relative to control cells. Each independent experiment is presented as a colored dot.

**Figure 8E** see figure legend , two-four independent experiments with the mean of more than 500 analysed cells presented as a colored dot.

**Figure 1-figure supplement 1A and Figure 4-figure supplement 1B-** see figure legend for details - mRNA analysis was from three independent experiments performed in triplicate samples

**Figure 4-supplement 1A, 1C, 1D, 1F and Figure 8-figure supplement 1-** see figure legend for details - Densitometry analysis to determine relative protein expression is presented relative to the control (100 %) from two-three independent experiments as indicated.

**Figure 7-figure supplement 2** – see figure legend for details – The % reduction of prestoblue was calculated from eight wells/ replicate experiment with three or four independent experiments performed. Each biological replicate for each independent experiment was colored coded.

**Statistical reporting**

- Statistical analysis methods should be described and justified
- Raw data should be presented in figures whenever informative to do so (typically when N per group is less than 10)
- For each experiment, you should identify the statistical tests used, exact values of N, definitions of center, methods of multiple test correction, and dispersion and precision measures (e.g., mean, median, SD, SEM, confidence intervals; and, for the major substantive results, a measure of effect size (e.g., Pearson's r, Cohen's d)
- Report exact p-values wherever possible alongside the summary statistics and 95% confidence intervals. These should be reported for all key questions and not only when the p-value is less than 0.05.

Please outline where this information can be found within the submission (e.g., sections or figure legends), or explain why this information doesn’t apply to your submission:

**Figure 1 -** Three replicates from matched WT and cavin3 KO HeLa cells were analysed with a total of 4206 proteins were robustly quantified, detected with >2 unique peptides and an FDR <1.0 % in at least 2 out of 3 replicate sas described in the results section (p.6) and in the corresponding figure legend.

**Figure 2 and Figure 2-figure supplement 1 -see Single molecule spectrometry in Material and Methods section for details –** more than 1000 events were collected in all cases.

**Figure 3F, 5E, 6E-H, Figure 3-figure supplement 1, Figure 6-figure supplement 1, Figure 8- figure supplement 2 -** see figure legend for details**-** Quantified PLA signals are represented as the number of red dots in 150 total cells (n=50 cells per experiment, three independent experiments as indicated. Significance was calculated using nested ANOVA, * p<0.05, ** p<0.01, ***p<0.001, ****p<0.0001

.

**Figure 7A, D-F**, **Figure 7-figure supplement 1-** see figure legend for details - LDH release - was calculated relative to control cells. Each independent experiment was colored coded and is presented as the Mean  ± SD black bars using a one-way ANOVA and Bonferroni’s multiple comparison test, * p<0.05, ** p<0.01, ***p<0.001, ****p<0.0001

**Figure 8E** see figure legend , two-four independent experiments with the mean of more than 500 analysed cells presented as a colored dot as indicated. Significance was calculated using one-way ANOVA and Bonferroni’s multiple comparison test, * p<0.05, ** p<0.01, ***p<0.001, ****p<0.0001

**Figure 1-figure supplement 1A and Supplementary Figure 5B-** see figure legend for details - mRNA analysis from three independent experiments performed in triplicate samples presented as the Mean ± SD black bars using a Student t-test. * p<0.05, ** p<0.01, ***p<0.001, ****p<0.0001

**Figure 4-figure supplement 1A, 1C, 1D, 1F and Figure 11-** see figure legend for details - Densitometry analysis to determine relative protein expression is presented relative to the control (100 %) from two-three experiments as indicated. Significance was calculated using one-way ANOVA and Bonferroni’s multiple comparison test) * p<0.05, ** p<0.01, ***p<0.001, ****p<0.0001

**Figure 7-figure supplement 2A-D and Figure 8-figure supplement 2C** – see figure legend for details – The % reduction of Prestoblue was calculated from eight wells / replicate experiment with three independent experiments were performed. Each replicate experiment was colored coded. Significance was calculated using a nested ANOVA, * p<0.05, ** p<0.01, ***p<0.001, ****p<0.0001

**Figure 8-figure supplement 2A-C** – see figure legend for details – Colonies larger than 50 cells were counted from each well of a 6 well plate. Each dot represents an individual well.

(For large datasets, or papers with a very large number of statistical tests, you may upload a single table file with tests, Ns, etc., with reference to sections in the manuscript.)

**Group allocation**

- Indicate how samples were allocated into experimental groups (in the case of clinical studies, please specify allocation to treatment method); if randomization was used, please also state if restricted randomization was applied
- Indicate if masking was used during group allocation, data collection and/or data analysis

Please outline where this information can be found within the submission (e.g., sections or figure legends), or explain why this information doesn’t apply to your submission:

Group allocation is not applicable to this manuscript as it does not contain clinical studies.

**Additional data files (“source data”)**

- We encourage you to upload relevant additional data files, such as numerical data that are represented as a graph in a figure, or as a summary table
- Where provided, these should be in the most useful format, and they can be uploaded as “Source data” files linked to a main figure or table
- Include model definition files including the full list of parameters used
- Include code used for data analysis (e.g., R, MatLab)
- Avoid stating that data files are “available upon request”

Please indicate the figures or tables for which source data files have been provided:

The source data file underlying the proteomics component of this study is provided in Supplementary File 1. Raw proteomics data will be uploaded to PRIDE upon publication. The source data underlying this study that includes raw western data with molecular weight standards is provided in the Figure 4-source data 1-5, Figure 8-sourced data 1 and 2, Figure 1-figure supplement source data 1-3, Figure 4-figure supplement 1 source data 1, Figure 4-figure supplement 2 source data 1 and 2, Figure 8-figure supplement 2 source data 1.
